# Immune characterization of lupus nephritis patients undergoing dialysis^☆^

**DOI:** 10.1016/j.jtauto.2025.100290

**Published:** 2025-05-02

**Authors:** Quentin Simon, François Gaillard, John Tchen, Delphine Bachelet, Karim Sacré, Katell Peoc'h, Noémie Jourde-Chiche, Eric Daugas, Nicolas Charles

**Affiliations:** a Université Paris Cité, Centre de recherche sur l'inflammation, INSERM UMR1149, CNRS EMR8252, Laboratoire d’Excellence Inflamex, Paris, France; b Inovarion, Paris, France; c Department of Nephrology, Hôpital Bichat, Assistance Publique-Hôpitaux de Paris, Paris, France; d Department of Internal Medicine, Hôpital Bichat, Assistance Publique-Hôpitaux de Paris, Paris, France; e Department of Biostatistical Epidemiology and Clinical research, Hôpital Bichat, Assistance Publique-Hôpitaux de Paris, INSERM CIC-EC 1425, Paris, France; f Université Paris Cité, Centre de Recherche sur l’Inflammation, INSERM UMR1149, Laboratoire d'Excellence GR-Ex, Paris, France; g Service de Biochimie, Hôpital Bichat, DMU BIOGEM, Assistance Publique-Hôpitaux de Paris, Paris, France; h Aix-Marseille Université, C2VN, INSERM, INRAE, Marseille, France; i Assistance Publique-Hôpitaux de Marseille, Centre de Néphrologie et Transplantation Rénale, Hôpital de la Conception, Marseille, France

**Keywords:** Systemic lupus erythematosus, Lupus nephritis, End stage kidney disease, Dialysis, Immunophenotyping, Disease activity

## Abstract

Systemic lupus erythematosus (SLE) activity decreases in some patients with end-stage kidney disease (ESKD). The impact of ESKD on the immune cell profile of SLE patients and lupus activity remains unclear. In this study, we aimed at characterizing immunologically inactive and active SLE patients undergoing dialysis therapy. Based on multi-parametric flow cytometry assays, an extensive immunophenotyping was performed on blood samples from 47 SLE patients undergoing hemodialysis, 10 non-dialyzed SLE patients with active lupus nephritis (aLN), 6 non-dialyzed patients with a history of LN currently in remission (rLN), and 20 healthy volunteers (HV) as controls (ClinicalTrials.gov Identifier: NCT03921398). The hemodialysis group was composed of 16 SLE patients with inactive disease (iHD), 22 with sustained low disease activity with a non-renal SLEDAI ≤4 (aHD≤4), and 9 highly active SLE patients (aHD>4). A factorial discriminant analysis was performed to validate the association between immune cell signatures and lupus activity. By compiling 12 cellular variables, we describe immune profiles related to highly active SLE patients or associated with both inactive and low-disease activity groups. As non-dialyzed active SLE patients, active patients undergoing hemodialysis showed a specific combination of increased numbers of circulating CD19^hi^ CD27^–^ “atypical naive” B cells, plasmablasts, CD16^+^ inflammatory monocytes and a basopenia. This study brings a comprehensive overview of immune cell signatures observed in SLE patients undergoing dialysis. We propose a simple immunophenotypic approach for the assessment of lupus activity that may provide help to data-driven personalized medicine in hemodialyzed SLE patients.

## Highlights

• SLE activity is considered to decline after initiation of maintenance dialysis.
• Clinical support for lupus activity assessment and therapeutic decision.
• Validation of previously identified key cellular pathogenic players.
• 4 leukocyte subsets assess lupus activity in hemodialyzed SLE patients.
• Applicable to data-driven personalized medicine for SLE patients with ESKD.

## 1 Introduction

Systemic lupus erythematosus (SLE) is a complex autoimmune disease of multifactorial etiology mainly affecting female of childbearing age. SLE is characterized by autoreactive antibodies raised against nuclear antigens which deposits and consequent inflammation can affect different organs including joints, skin, central nervous system and kidneys [1]. Lupus nephritis (LN) is observed in ∼30–50 % of SLE patients and represents one of the most severe manifestations of SLE [1]. Five to 20 % of LN patients develop end-stage kidney disease (ESKD) within 10 years after SLE diagnosis, requiring renal replacement therapies (RRT) [1,2].

Immune dysfunction and chronic inflammation in SLE are promoted by innate and/or adaptive immune cells, including dendritic cells, monocytes and macrophages, neutrophils, basophils as well as B and T lymphocytes [[3], [4], [5], [6]]. SLE is characterized by B cell hyperactivity with an overrepresentation in the blood of activated naive (acN: CD19^hi^ IgD ^+^ CD27^–^) B cells, double negative (DN: CD19^+^ IgD^–^ CD27^–^) B cells and plasmablasts (CD19^lo^ CD27^hi^) along with autoantibody production by terminally differentiated B cells [6]. Both acN and DN B cell populations show enrichment for autoreactive cells and are precursors of antibody-secreting cells [7,8].

Monocytes from SLE patients have a reduced ability in the clearance of apoptotic cell debris, display altered pro-inflammatory secretory functions, and the frequency of circulating CD16^+^ monocytes positively correlates with SLE disease activity and anti-dsDNA autoantibody titers [5]. Neutrophilic and basophilic granulocytes are also key players in the SLE pathogenesis [[9], [10], [11]]. Dysregulated neutrophils have increased neutrophil extracellular traps (NET, NETosis) and pro-inflammatory mediator release abilities [10]. Basophil activation and basopenia are associated with SLE disease activity and LN [4,12,13]. Basophils preferentially migrate toward secondary lymphoid organs (SLO) during SLE, where they sustain B cell differentiation and autoantibody production through T follicular helper (TFH) cell promotion, resulting in increased IC deposits in the kidneys [4,12,14].

Activity of SLE is subdued among a substantial fraction of patients who progressed to ESKD and are on chronic RRT [[15], [16], [17]]. The biological and immunological processes involved in this decline of SLE activity need to be further investigated to help clinicians adjust treatment and identify new regulatory mechanisms that could be translated into therapeutic opportunities for lupus patients [18]. However, whether the immunological profiles of active and inactive SLE patients undergoing dialysis reflect their disease activity and their need of maintenance therapy, as in LN patients before ESKD is reached, needs to be addressed.

The present study aimed at identifying immune profiles associated with disease activity in SLE patients undergoing hemodialysis (HD). By focusing on cell populations known to be associated with SLE activity, we propose a simple cellular assessment defining the immunological activity status of a given SLE patient (including 10.13039/100004792HD patients) that could be useful to support clinical evaluation.

## 2 Methods

### 2.1 Patients and healthy volunteers

Our study is part of the ELUDIAL project dedicated to the identification of new therapeutic targets for SLE (ClinicalTrials.gov Identifier: NCT03921398), by comparing molecular and cellular particularities of inactive and active SLE patients undergoing dialysis. The ELUDIAL cohort included patients from the Ile-de-France area treated with maintenance hemodialysis (HD) because of chronic kidney disease (CKD) stage 5 due to lupus nephritis and ≥18 years old. Patients with a HD vintage inferior to 9 months after graft loss, with active infection or allergy, pregnant or breastfeeding women were excluded.

The study was performed transversally during a 2-h examination at the Nephrology Outpatient Clinic of the Bichat Hospital. Patients on dialysis were included and examined on a non-dialysis day. The following were recorded and performed: general clinical examination, evaluation of lupus activity to calculate the SELENA-SLEDAI score and non-renal SELENA-SLEDAI score (Safety of Estrogens in Lupus Erythematosus National Assessment SLEDAI) [17,19], current treatment, treatment change within the last 6 months and blood sampling. Non-renal SELENA-SLEDAI score (nrSLEDAI) was calculated by removing urinary casts, hematuria, proteinuria and pyuria parameters from SELENA-SLEDAI index.

The ELUDIAL cohort included 47 SLE patients undergoing HD. Ten patients with active LN without RRT (aLN) were added as positive controls of lupus activity: all were ≥18 years old with biopsy-proven active LN, and naive of immunosuppressive therapy and glucocorticoids. Twenty healthy volunteers (HV) matched for age and sex with HD patients were included as negative controls. HV had no history of autoimmune disease, were not receiving immunosuppressive therapy nor glucocorticoids, and were neither pregnant nor breastfeeding. Six non-dialyzed patients with a history of LN but currently in remission (rLN) were included in this study, as controls. HD SLE patients were divided into two groups according to disease activity. The active HD patient group (aHD) included 31 patients with non-renal (nr) SLE disease activity index (SLEDAI) score >0 at inclusion or during the last 18 months. The inactive HD (iHD) patients group included 16 individuals with nrSLEDAI = 0 at inclusion and during the last 18 months, among which 11 were considered off-treatment (without immunosuppressive treatments and prednisone ≤ 5 mg/day) and 5 were under treatment at inclusion (corticosteroids > 5 mg/day or mycophenolate mofetil or azathioprine). Patients’ demographic and clinical characteristics are summarized in Table 1. Individual characteristics of HD SLE patients are indicated in Supplementary Table S1.

**Table 1:** **Demographic and clinical characteristics of SLE patients and healthy volunteers.** SLE: Systemic Lupus Erythematosus; HD: Hemodialysis; LN: Lupus Nephritis; aLN: active LN patients; rLN: SLE patients with a history of LN but currently in remission; M: Male; F: Female; IQR: Interquartile range; SLEDAI: SLE Disease Activity Index; NA: Not Applicable; MD: Missing Data.

	HD patients (n = 47)		non-HD patients		Healthy
	Active (aHD)	Inactive (iHD)	Active (aLN)	Inactive (rLN)	Volunteers (HV)
	n = 31	n = 16	n = 10	n = 6	n = 20
**DEMOGRAPHY**					
Gender, n (%)					
M	5 (16 %)	4 (25 %)	2 (20 %)	0 (0 %)	5 (25 %)
F	26 (84 %)	12 (75 %)	8 (80 %)	6 (100 %)	15 (75 %)
Age, median (IQR)	40.0 (34.6, 50.9)	49.5 (42.2, 63.4)	30.8 (29.0, 34.8)	44.6 (32.7, 52.0)	42.4 (36.1, 50.0)
**LUPUS ACTIVITY AT INCLUSION**					
Time since last lupus flare-up (years), median (IQR)	1.6 (1.0, 6.9)	7.1 (4.6, 11.7)	0.1 (0.0, 0.1)	11.5 (7.8, 19.2)	NA
"Off-treatment" for at least 18 months before inclusion, n (%)	10 (32 %)	11 (69 %)	0 (0 %)	3 (50 %)	NA
Clinical extinction for at least 18 months before inclusion, n (%)	12 (39 %)	16 (100 %)	0 (0 %)	6 (100 %)	NA
non-renal SLEDAI, median (IQR)	4.0 (2.0, 5.5)	0.0 (0.0, 0.0)	13.0 (10.5, 15.5)	0 (0.0, 0.5)	NA
**DIALYSIS PARAMETERS**					
Time from dialysis initiation (years), median (IQR)	2.7 (1.7, 4.0)	4.2 (1.0, 8.4)	NA	NA	NA
Type of dialysis, n (%)					
Hemodialysis	28 (90 %)	11 (69 %)	NA	NA	NA
Hemodiafiltration	3 (10 %)	5 (31 %)	NA	NA	NA
**SEROLOGY**					
Low complement, n (%)	21 (68 %)	0 (0 %)	9 (90 %)	0 (0 %)	NA
Positive anti-native DNA antibodies, n (%)	15 (48 %)	0 (0 %)	8 (80 %)	1 (17 %)	NA
Positive antinuclear antibodies, n (%)	9 (30 %) MD: 1	2 (13 %)	4 (40 %)	MD	NA
**LUPUS TREATMENT**					
Hydroxychloroquine, n (%)	20 (65 %)	8 (50 %)	7 (70 %)	6 (100 %)	0 (0 %)
Steroids, n (%)	23 (74 %)	9 (56 %)	3 (30 %)	4 (67 %)	0 (0 %)
Immunosuppressive treatment, n (%)	10 (32 %)	2 (12 %)	2 (20 %)	1 (17 %)	0 (0 %)
Biological therapy, n (%)	2 (6 %)	0 (0 %)	0 (0 %)	0 (0 %)	0 (0 %)
Antibiotics, n (%)	4 (13 %)	3 (19 %)	0 (0 %)	0 (0 %)	0 (0 %)

## 4 Results

### 4.1 Simple identification of two B cell populations associated with SLE activity

**Fig. 1:**
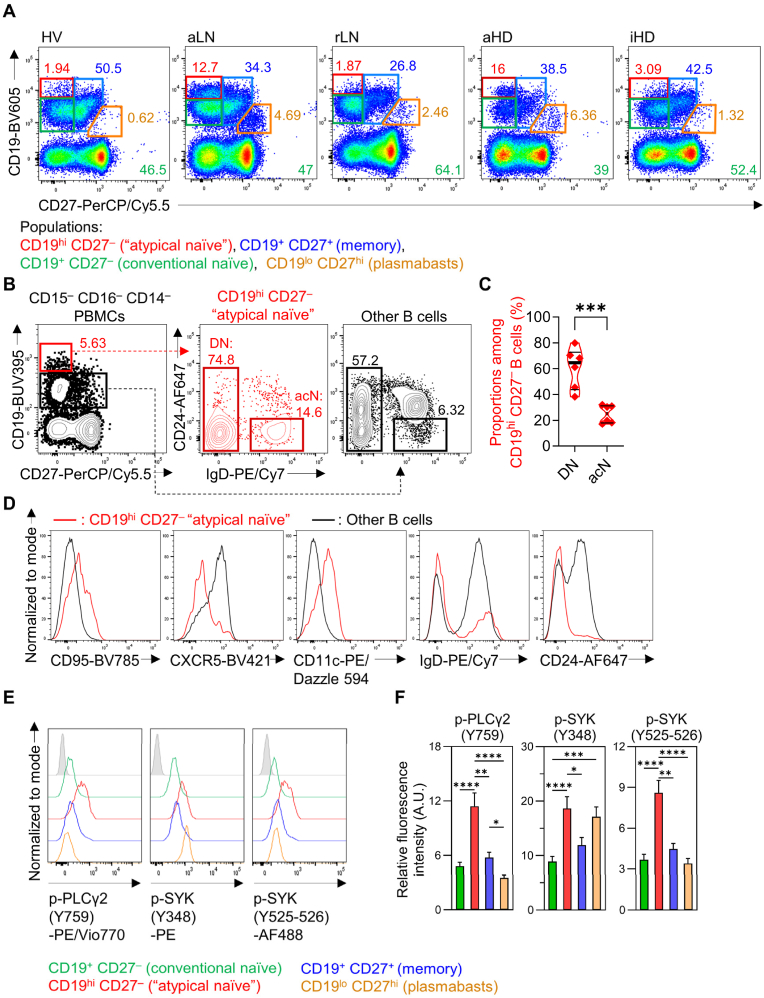
**Identification and characterization of CD19^hi^ CD27^–^ ‘’atypical naive’’ B cells in SLE patients.** (**A**) Identification of B cell subsets using CD19 and CD27 markers (represented in SSC^lo^ CD16^–^ CD14^–^ leukocytes). Flow cytometry plots are representative of HV (healthy volunteers), aLN (active SLE patients with lupus nephritis (LN)), rLN (SLE patients with a history of LN currently in remission), aHD (active patients undergoing hemodialysis (HD)) and iHD groups (inactive patients undergoing HD). Proportions (%) of subsets among CD19^+^ B cells are indicated in green: CD19^+^ CD27^–^ conventional naive, blue: CD19^+^ CD27^–^ memory, red: CD19^hi^ CD27^–^ ‘’atypical naive”, orange: CD19^lo^ CD27^hi^ plasmablasts). (**B**) Representative flow cytometry plots of IgD and CD24 expression in CD19^hi^ CD27^–^ ‘’atypical naive’’ B cells (red gate with proportions among CD19^+^ B cells) and other B cells (black gate), in CD15^–^ CD16^–^ CD14^–^ Peripheral Blood Mononuclear Cells (PBMCs). Proportions (%) of IgD^+^ CD24^–^ activated naive (acN) and IgD^–^ CD27^–^ double negative (DN) within ‘’atypical naive’’ (middle red panel) or other (right black panel) B cells are indicated above gates. (**C**) DN and acN among CD19^hi^ CD27^–^ B cells (%). (**D**) Flow cytometry histograms of CD95, CXCR5, CD11c, IgD and CD24 expression in CD19^hi^ CD27^–^ (red line) and other B cells (black line). (**E**) Phosphorylation levels of PLCγ2 (Y759) and SYK (Y348 and Y525-526) measured by flow cytometry in 4 B cell populations of active lupus patients. Filled grey: isotype control. (**F**) Relative fluorescence intensity of PLCγ2 and SYK phosphorylations in the 4 B cell subsets in aHD patients (n = 31), mean +s.e.m. Arbitrary Unit (A.U.): ratio between geometric mean fluorescence intensity of specific staining on isotype controls. (**B**–**D**) Data are representative of on the analysis of PBMCs from 6 active SLE patients. (**C**) Data are presented as individual values in truncated violin plots showing median (thick line) and quartiles (thin lines). ∗∗∗:*P* ≤ 0.001 by unpaired *t*-test. (**F**) Data are presented as mean +s.e.m. ∗*P* ≤ 0.05, ∗∗*P* ≤ 0.01, ∗∗∗*P* ≤ 0.001, ∗∗∗∗*P* ≤ 0.0001, by Kruskal-Wallis test followed by Dunn's post-tests.

To identify the most relevant immune cell populations associated with the disease activity in SLE patients undergoing hemodialysis (HD), we performed an immunophenotyping of biopsy-proven active non-dialyzed patients with lupus nephritis (aLN) (positive controls), SLE patients with a history of LN currently in remission (rLN) and healthy volunteers (HV) (negative controls), and compared them to our HD patient samples (Table 1). Concerning B cells, we used a simple flow cytometry gating strategy and quantified 4 relevant B cell populations previously described as being dysregulated subsets in active SLE patients: CD19^hi^ CD27^–^ (“atypical naive” B cells, see below), CD19^+^ CD27^+^ (memory B cells), CD19^+^ CD27^–^ (conventional resting naive B cells), and CD19^lo^ CD27^hi^ (plasmablasts) (Fig. 1A) [7,8].

**Fig. 2:**
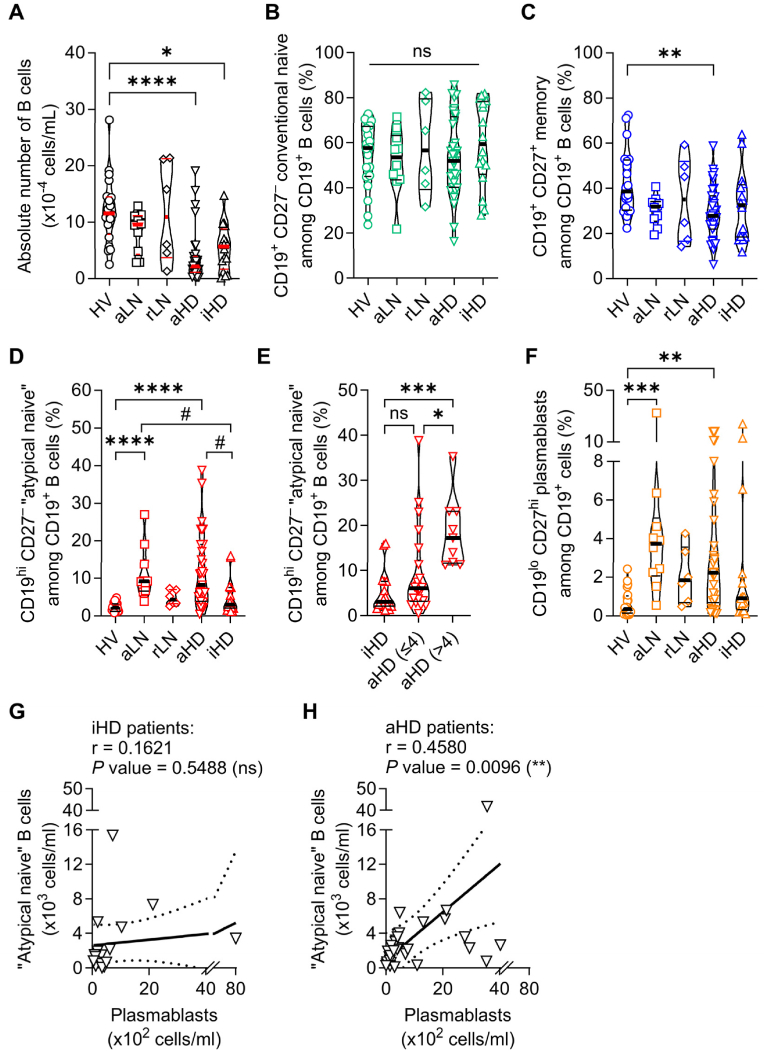
**Quantification of 4 relevant B cell populations in SLE patients.** (**A**) B cell counts in HV (n = 20), aLN (n = 10), rLN (n = 6), aHD (n = 31) and iHD (n = 16) groups. (**B**-**D,F**) Frequencies (%) of subsets defined in Fig. 1A among B cells in HV, aLN, rLN, aHD and iHD. (**E**) CD19^hi^ CD27^–^ (%) among B cells in aHD≤4 (aHD with nrSLEDAI ≤4) (n = 22), aHD>4 (nrSLEDAI >4) (n = 9) and iHD (n = 16) patients. (**G**,**H**) Correlation (and linear regression with 95 % confidence intervals (dotted lines)) between CD19^hi^ CD27^–^ B cells and plasmablasts absolute numbers in iHD (**G**, Pearson's r = 0.1621, *P* = 0.54, n = 16) and aHD patients (**H**, Pearson's r = 0.4580, *P* = 0.0096, n = 31). (**A**–**F**) Data are presented as individual values in truncated violin plots showing median (thick line) and quartiles (thin lines). ∗*P* ≤ 0.05, ∗∗*P* ≤ 0.01, ∗∗∗*P* ≤ 0.001, ∗∗∗∗*P* ≤ 0.0001, ns = not significant, by one-way ANOVA followed by Tukey's post-tests (**B**,**C**), or by Kruskal-Wallis tests followed by Dunn's post-tests (**A**,**D**-**F**). (**D**) # (aLN *vs* iHD): *P* = 0.0737, # (aHD *vs* iHD): *P* = 0.0794. Pairwise comparisons not displayed on graphs had *P* values > 0.1.

Autoantibody secreting cells are mainly derived from acN (CD19^hi^ IgD ^+^ CD27^–^) and DN2 (IgD^–^ CD27^–^ CXCR5^–^ CD11c^+^) B cells that are enriched in the 9G4 idiotope (VH4-34 immunoglobulin gene rearrangement) and are known to expand in active SLE patients [7,8]. The simply gated CD19^hi^ CD27^–^ “atypical naive” cells are mainly composed of both DN2 and acN B cells (Fig. 1B–D) and are the most activated B cell subset (with plasmablasts) as evidenced by the high phosphorylation status of phospholipase C gamma 2 (PLCγ2) and spleen tyrosine kinase (SYK) proteins measured by intracellular flow cytometry in this B cell subset (Fig. 1E,F and Fig. S1A).

Patients undergoing HD had a significant reduction in total circulating B cell numbers compared to HV (Fig. 2A). While no difference was observed in the frequency of conventional naive B cells among CD19^+^ cells between groups, active SLE patients on hemodialysis (aHD) had a significant reduction of CD27^+^ memory B cell frequency compared to HV (Fig. 1, Fig. 2B,C). CD19^hi^ CD27^–^ “atypical naive” B cells were very rare in HV, rLN and inactive HD (iHD) individuals while their proportions within B cells were dramatically increased in both aHD and aLN patients, suggesting their association with disease activity independently of the dialysis status (Fig. 1, Fig. 2D). This point was further confirmed when we separated aHD individuals into two groups (nrSLEDAI ≤4 *vs* > 4) referring to the lupus low disease activity state (LLDAS) SLEDAI item [20]. Indeed, the frequency of CD19^hi^ CD27^–^ cells within B cells was significantly increased in HD patients with nrSLEDAI >4 (aHD>4), in comparison to aHD≤4 and inactive patients on hemodialysis (iHD) (Fig. 2E). Some aLN and aHD patients exhibited elevated levels of CD19^lo^ CD27^hi^ plasmablasts (Fig. 1, Fig. 2F), but iHD, rLN and HV individuals mostly shared a common B cell phenotype (Fig. 1, Fig. 2B-D,F). Interestingly, CD19^hi^ CD27^–^ B cells and plasmablasts numbers were positively correlated in aHD but not in iHD patients (Fig. 2G and H).

Overall, these results indicated that the accumulation of hyperactivated CD19^hi^ CD27^–^ “atypical naive” B cells, easily identified, was associated with SLE disease activity in lupus patients undergoing hemodialysis as in non-dialyzed patients. Elevated number of “atypical naive” B cells may reflect the increased representation of hyperactivated autoreactive B cells in active SLE patients that may further differentiate into antibody-secreting cells (plasmablasts) which are as well overrepresented in active patients.

Of note, a simple T cell analysis did not provide a way to distinguish inactive from active HD patients. Indeed, except in rLN group, CD4^+^ T cell lymphocytopenia was observed in all the other SLE patients analyzed. CD8α^+^ T cell numbers in blood were equivalent to HV individuals for all SLE patient groups (Fig. S1B–D).

### 4.2 Circulating basophil numbers reflect disease activity, unlike neutrophil and eosinophil numbers

**Fig. 3:**
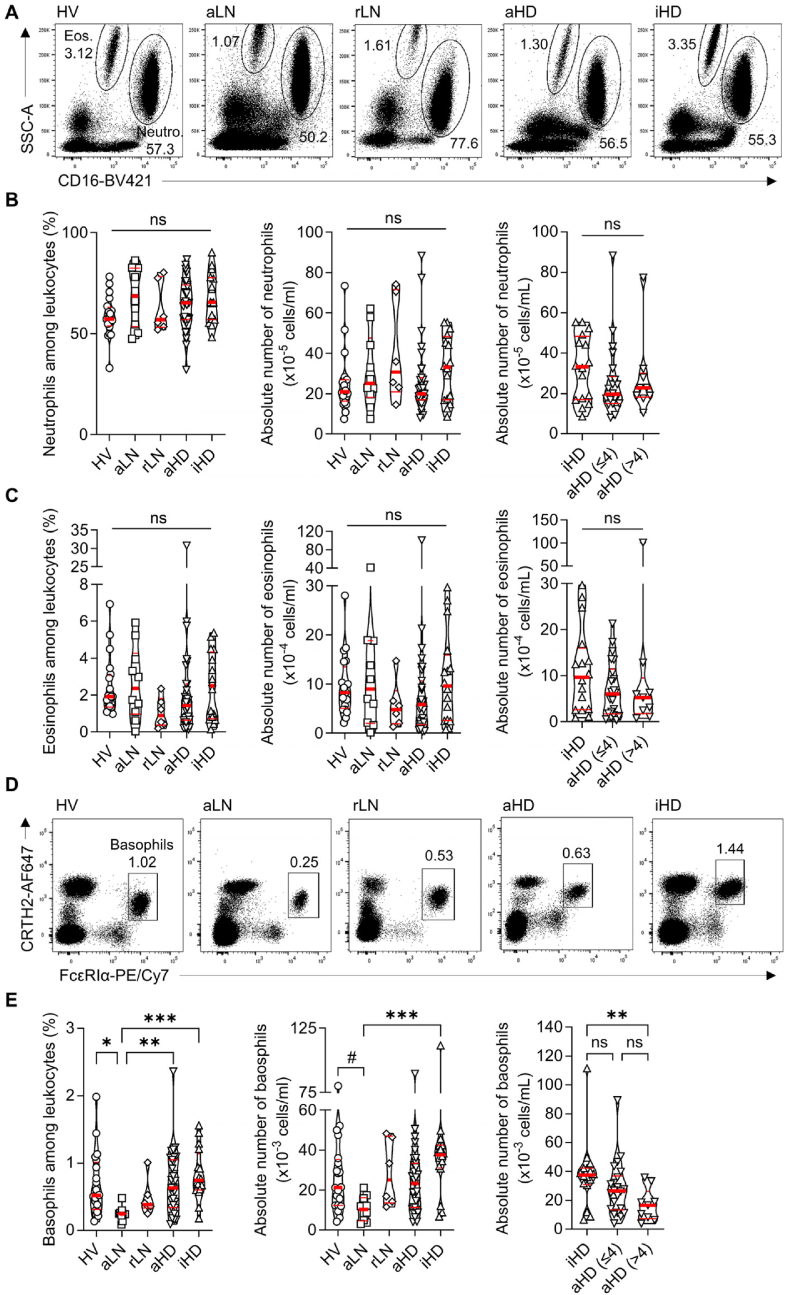
**Granulocytes in SLE patients undergoing hemodialysis.** (**A**) Representative flow cytometry plots of eosinophils (Eos.) and neutrophils (Neutro.) selection by flow cytometry, based on side scatter (SSC-A) parameter and expression of CD16 molecule. Proportions (%) of Eos. and Neutro. among leukocytes are indicated for HV, aLN, rLN, aHD and iHD individuals. Proportions (%) and absolute numbers of neutrophils (**B**) and eosinophils (**C**) among leukocytes in HV (n = 20), aLN (n = 10), rLN (n = 6), aHD (n = 31) and iHD (n = 16) groups. Neutrophil (**B**, right panel) and eosinophil (**C**, right panel) counts in iHD (n = 16), aHD≤4 (n = 22), and aHD>4 (n = 9) individuals. (**D**) Representative flow cytometry plots of basophil gating based on CRTH2 and FcεRIα expression (in CD14^–^, CD16^–^, CD19^–^ leukocytes). (**E**) Proportions (%) among leukocytes and absolute numbers of basophils in HV, aLN, rLN, aHD and iHD groups. Right panel: basophil counts in iHD, aHD≤4, and aHD>4 patients. (**B**,**C**,**E**) Data are presented as individual values in truncated violin plots showing median (thick red line) and quartiles (thin red lines). ∗*P* ≤ 0.05, ∗∗*P* ≤ 0.01, ∗∗∗*P* ≤ 0.001, ns = not significant by Kruskal-Wallis tests followed by Dunn's post-tests. (**E**) # (HV *vs* aLN): *P* = 0.0747. Pairwise comparisons not displayed on graphs were ns.

Concerning granulocytes, no difference in blood neutrophil nor eosinophil frequencies and numbers were noticed between the five groups of individuals (Fig. 3A–C). Accumulation of basophils in SLO is associated with peripheral basopenia and correlates with SLE disease activity [4,12]. As expected, circulating basophil frequency and numbers were drastically reduced in aLN patients when compared to HV subjects (Fig. 3D and E). iHD patients showed a normal number of basophils unlike aHD patients who displayed an intermediate phenotype between aLN and inactive patients (Fig. 3E). Basophil counts were significantly lower in highly active HD patients (nrSLEDAI >4) compared to iHD group while it was not the case for aHD patients with nrSLEDAI ≤4 (Fig. 3E). Thus, among granulocyte populations, basophils were the only population which variation in their circulating numbers (basopenia) was clearly associated with disease activity. This peripheral basopenia reflects the accumulation of basophils in SLO, where they support autoantibody production through their help to pathogenic TFH and B cells [4,[12], [13], [14]].

**Fig. 4:**
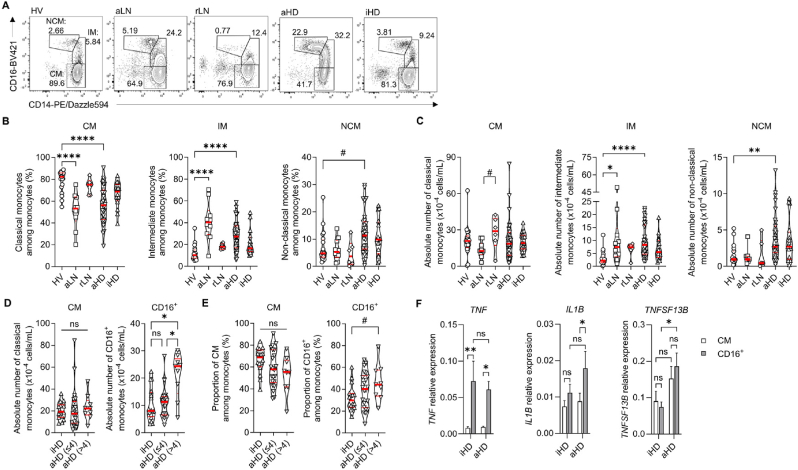
**Analysis of monocyte subsets in healthy volunteers and lupus patients.** (**A**) Representative flow cytometry plots of CD14 and CD16 expression in CD19^–^, CD4^–^, CD8α^–^ and not SSC^hi^ leukocytes. Proportions (%) of Non-Classical Monocytes (NCM), Intermediate Monocytes (IM) and Classical Monocytes (CM) among monocytes are indicated next to the corresponding gates for HV, aLN, rLN, aHD and iHD individuals. Proportions (%) among monocytes (**B**) and absolute numbers (**C**) of CM, IM and NCM in HV (n = 20), aLN (n = 10), rLN (n = 6), aHD (n = 31) and iHD (n = 16) individuals. Absolute numbers (**D**) and proportions among monocytes (**E**) of CM and CD16^+^ monocytes in iHD (n = 16), aHD≤4 (n = 22), and aHD>4 (n = 9) SLE patients. (**F**) *TNF*, *IL1B* and *TNFSF13B* mRNA expression levels relative to GAPDH mRNA (2^–ΔCt^) measured by real-time PCR in sorted classical (CM, white bars) and CD16^+^ (grey bars) monocytes from iHD (n = 7) and aHD>4 (n = 7) patients (mean +s.e.m). (**B-E**) Data are presented as individual values in truncated violin plots showing median (thick red line) and quartiles (thin red lines). Statistical analyses were by Kruskal-Wallis tests followed by Dunn's post-tests (**B-D**), or by one-way ANOVA followed by Tukey's post-tests (**E**). (**F**) Two-way ANOVA followed by uncorrected Fisher's tests. ∗*P* ≤ 0.05, ∗∗*P* ≤ 0.01, ∗∗∗*P* ≤ 0.001, ∗∗∗∗*P* ≤ 0.0001, ns = not significant. (**B**) # (HV *vs* aHD) *P* = 0.0629, (**C**) # (aLN *vs* rLN): *P* = 0.0931, (**E**) # (iHD *vs* aHD>4): *P* = 0.0826. Pairwise comparisons not displayed on graphs were ns.

**Fig. 5:**
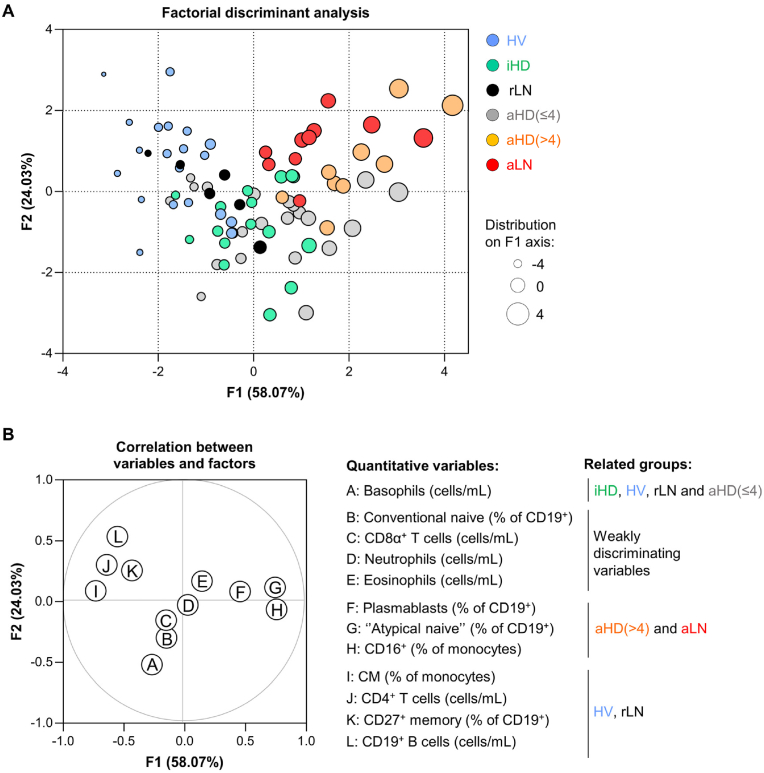
**Immunophenotyping based discriminant analysis of healthy controls and lupus patients.** (**A**) Factorial Discriminant Analysis (FDA) was performed using the following 12 quantitative variables: absolute numbers of basophils, neutrophils, eosinophils, CD8α^+^ T cells, CD4^+^ T cells, CD19^+^ B cells, proportions among CD19^+^ cells of conventional naive B cells, memory B cells, “atypical naive” B cells and plasmablasts, and proportions among monocytes of classical (CM) and CD16^+^ monocytes. F1 and F2 factors show the efficiency of FDA in discriminating HV (n = 20, blue dots), iHD (n = 16, green dots), aHD≤4 (n = 22, grey dots), aHD>4 (n = 9, orange dots), rLN (n = 6, black dots) and aLN (n = 10, red dots) individuals. The sizes of the dots reflect their distribution on the F1 axis. (**B**) 12 quantitative variables (A–L) used to perform FDA and their correlation with F1 and F2 axis. Related groups column summarizes the association between quantitative variables and their efficiency in stratifying patients depending on the predefined group membership.

### 4.3 Increased CD16^+^ inflammatory monocyte counts are characteristic of active SLE individuals

Monocytes can be divided into three subsets depending on CD14 and CD16 expression: CD14^hi^ CD16^–^ classical monocytes (CM), CD14^hi^ CD16^+^ intermediate monocytes (IM) and CD14^lo^ CD16^++^ non-classical monocytes (NCM) (Fig. 4A). Compared to CM, CD16^+^ monocytes (NCM + IM) are considered as inflammatory effectors [5]. An alteration of the monocyte compartment was observed in both aLN and aHD SLE patients, illustrated by the increased proportion of IM and the decreased proportion of CM among total monocytes when compared to HV (Fig. 4B). Although no significant difference was observed for CM counts between patient groups (Fig. 4C), the frequencies among leukocytes and absolute numbers of both NCM and IM were increased in aHD patients compared to HV controls (Fig. 4C and S1E). IM were also overrepresented in aLN compared to HV group (Fig. S1E). Remarkably, the numbers of CD16^+^ monocytes (IM + NCM) were increased in aHD>4 patients compared to iHD and aHD≤4, while CM counts did not differ between iHD, aHD≤4 and aHD>4 groups (Fig. 4D). CD16^+^ frequency among monocytes tended to increase between iHD and aHD>4 patients, although not significantly (*P* = 0.0826, Fig. 4E).

Since considering only the CD16 positivity of the monocyte populations was sufficient to separate active HD patients from the less or non-active HD groups, we validated the inflammatory profile of CD16^+^ monocytes in HD patients by sorting CD16^–^ CM and CD16^+^ monocytes from iHD and aHD blood samples (Fig. S2). We determined that CD16^+^ monocytes from both iHD and aHD patients highly expressed the Tumor Necrosis Factor (*TNF*) gene compared to CM (Fig. 4F). In addition, CD16^+^ monocytes isolated from aHD individuals had more IL-1β transcripts than their corresponding CM unlike the ones isolated from iHD patients (Fig. 4F). Importantly, monocytes from aHD group showed higher levels of the B-cell activating factor of the *TNF* superfamily (BAFF) mRNA (encoded by the *TNFSF13B* gene) compared to iHD patients, especially in CD16^+^ monocytes (Fig. 4F).

Altogether, these data demonstrated that increased numbers of circulating CD16^+^ inflammatory monocytes were associated with SLE activity in HD patients which may reflect the pro-inflammatory and B cell pro-survival environments that promote autoantibody production in active SLE.

### 4.4 Immunophenotyping summary and stratification of SLE patients

The ELUDIAL cohort comprises 6 groups of individuals, undergoing dialysis (iHD, aHD≤4, aHD>4) or not (HV, aLN, rLN). HV, rLN and iHD individuals composed an inactive pool, whereas aHD>4 and aLN formed a highly active SLE group (Table 1).

To decipher the heterogeneity of our groups of patients we performed a factorial discriminant analysis (FDA), using 12 immune variables related to granulocytes, monocytes, B and T cells distribution previously described in this study. FDA showed that the combination of those 12 quantitative variables led to the identification of immune signatures associated with HV individuals, highly active SLE patients (aLN and aHD>4) or related to iHD, rLN and aHD≤4 groups who shared a similar immunophenotype (Fig. 5A). HV group was defined by normal proportions of circulating CD4^+^ T and B cells and CD27^+^ memory B cells that were all reduced in active SLE patient groups (Fig. 5B). Active SLE patients (aLN and aHD>4) were mainly characterized by the 4 following immune cell type modifications: increased proportions of CD16^+^ monocytes, plasmablasts and “atypical naive” B cells and basopenia. As expected from above results (Fig. 2, Fig. 3, Fig. 4 and S1), absolute number of CD8α^+^ T cells, neutrophils, eosinophils as well as the frequency of conventional naive B cells were weakly involved in patients’ stratification. Of note, we wondered whether dialysis therapy or treatments were significantly interacting with the disease activity status and could contribute to the observed immune phenotype. Inactive (iHD and rLN) and highly active (aHD>4 and aLN) SLE patients were sub-categorized based on their treatments or their hemodialysis status and two-way ANOVA analyses revealed that neither of these parameters were likely to have an effect on the 4 cell populations of main interest (Fig. S3). Disease activity was the only parameter contributing to the modifications of the immune signature (Fig. S3).

Of note, our approach did not allow the separation of iHD patients from those with a lower disease activity (aHD≤4). Nonetheless, this observation is consistent with one of the LLDAS attainment item (SLEDAI ≤4) [20] and suggests a common immune cell signature between inactive SLE patients and those with a low disease activity.

Overall, the FDA prediction method led us to conclude that CD19^hi^ CD27^–^ “atypical naive” B cell, CD16^+^ monocyte and plasmablast proportions, and absolute number of basophils were effective in discriminating inactive SLE individuals from most of highly active patients, undergoing hemodialysis (aHD>4) or not (aLN).

## 5 Discussion

SLE is a complex disease in which many immune cell populations have been described to be altered in terms of function and/or proportion. Heterogeneity of clinical manifestations and molecular signatures in SLE patients have raised the interest of stratifying patients to adapt treatments. Importantly, this clinical heterogeneity has also been reported in SLE patients with LN-related ESKD undergoing RRT [15,17]. Within the ELUDIAL cohort, we observed that a third of SLE patients undergoing hemodialysis had an inactive disease at time of inclusion. Similarly, Nossent et al. reported in a cohort of 55 patients with ESKD that approximately one third of active individuals (nrSLEDAI >0) reached SLE inactivity (nrSLEDAI = 0) after starting dialysis [17]. The immune phenotype of SLE patients undergoing dialysis has not been investigated before our present study. By taking advantage of this decline of SLE activity in some dialyzed patients, we designed a cellular assay to evaluate lupus activity. Albeit non-exhaustive, our study covered a large spectrum of immune cell populations.

We show that immune parameters known to be associated with SLE activity in non-dialyzed patients are also found in patients undergoing HD. In addition to basopenia and increased numbers of inflammatory monocytes, we reinforced the description of an abnormal B cell compartment in SLE patients. We performed a deep characterization of the simply identified CD19^hi^ CD27^–^ B cells and showed that they comprised both DN2 and acN B cells, and were hyperactivated regarding phosphorylation status of PLCγ2 and SYK proteins. Increased representation of these so-called “atypical naive” B cells was associated with plasmablasts accumulation, suggesting that these cells could give rise to autoreactive plasmablasts under the influence of the lupus environment. BAFF plays a critical role in the development of autoimmunity, including SLE, especially through its function on B cell survival, activation and maturation [21]. We showed that CD16^+^ monocytes from aHD patients overexpressed BAFF compared to those from iHD individuals. These results are in line with the fact that the increased number of inflammatory monocytes is associated with the survival and maturation of self-reactive B cells and related to plasmablasts overrepresentation in highly active SLE patient blood, including patients undergoing hemodialysis.

Limitations of our descriptive study include the relatively small number of patients in some groups of our cohort and may question our findings when no differences between group of patients are observed. Indeed, the FDA analysis did not identify a phenotypic profile specifically associated with lupus low disease activity (aHD≤4) allowing to distinguish them from iHD patients. Nevertheless, this observation is consistent with the reliability of extending the LLDAS status established in non-dialyzed lupus patients to lupus patients undergoing HD [20]. Furthermore, the similarity of the immune cell phenotype of aHD≤4, iHD and rLN patients may also be due to the fact that the contributors to the SELENA-SLEDAI score of aHD≤4 patients were mainly serological parameters (low complement and anti-dsDNA antibodies) and not clinical activity. Thus, some aHD≤4 patients should not only be considered as LLDAS patients but even as patients with a Serologically Active and Clinically Quiescent (SACQ) lupus, a disease status corresponding to an inactive disease [22]. We then interpreted the finding that aHD≤4 patients share a similar immune cell phenotype with iHD and rLN patients in a consistent manner. On the other hand, FDA bringing aLN patients near to aHD>4 group and rLN with iHD patients suggests that non-dialyzed lupus patients and those who undergo HD have close immune profiles.

The conclusions of the FDA put forward a combination of variables effective in separating SLE patients with a low or inactive disease from highly active individuals. Thus, we propose a simple immunophenotypic approach that may serve as an objective biomarker for the assessment of lupus activity in SLE patients, especially those with clinical manifestations that are not specific for lupus activity. This objectification could be assessed by applying a simple flow cytometry panel dedicated to the quantification of CD19^hi^ CD27^–^ “atypical naive” B cells, CD19^lo^ CD27^hi^ plasmablasts, basophils and CD16^+^ inflammatory monocytes. Further confirmation of this approach in independent cohorts will be required to validate the clinical benefits of such immune activity objectification for hemodialyzed and non-dialyzed SLE patients.

**Key messages**

**What is already known on this topic**

Systemic Lupus Erythematosus (SLE) activity has long been considered to decline after initiation of maintenance dialysis. However, if not appropriate, treatment withdrawal or continuation may favor SLE flares or increased risk of infection, respectively, impacting patient survival, quality of dialysis and quality of life.

**What this study adds**

This study describes the immunophenotype of key cellular pathogenic players from lupus nephritis patients with end-stage kidney disease (ESKD) undergoing hemodialysis with either inactive or active disease. Comparisons with healthy volunteers and patients with active or inactive lupus nephritis before ESKD allowed to describe 4 easily identifiable leukocyte subsets enabling the assessment of lupus activity in hemodialyzed patients.

**How this study might affect research, practice or policy**

This study brings a comprehensive overview of immune cell signatures observed in SLE patients undergoing dialysis and provide a simple immunophenotypic approach for the assessment of lupus activity applicable to data-driven personalized medicine.

## CRediT authorship contribution statement

**Quentin Simon:** Writing – review & editing, Writing – original draft, Project administration, Methodology, Investigation, Formal analysis. **François Gaillard:** Writing – review & editing, Project administration, Methodology, Investigation, Formal analysis, Conceptualization. **John Tchen:** Writing – review & editing, Methodology, Investigation, Formal analysis. **Delphine Bachelet:** Writing – review & editing, Methodology, Formal analysis, Data curation. **Karim Sacré:** Writing – review & editing, Investigation, Formal analysis, Data curation. **Katell Peoc'h:** Writing – review & editing, Methodology, Investigation. **Noémie Jourde-Chiche:** Writing – review & editing, Methodology, Investigation, Formal analysis, Conceptualization. **Eric Daugas:** Writing – review & editing, Writing – original draft, Supervision, Project administration, Methodology, Investigation, Funding acquisition, Formal analysis, Conceptualization. **Nicolas Charles:** Writing – review & editing, Writing – original draft, Supervision, Project administration, Methodology, Investigation, Funding acquisition, Formal analysis, Conceptualization.

## 3 Ethical approval

All patients and healthy volunteers were included between June 2019 and December 2021. The study was conducted in accordance with the principles of the Declaration of Helsinki. All individuals gave a written informed consent. The Comité de Protection des Personnes Ouest V (Rennes, France) approved the study on March 25th, 2019 (IDRCB no: 2018-A03072-53).

### 3.1 Experimental procedures

Antibodies, reagents, software, and equipment are listed in Supplementary Table S2.

### 3.2 Human samples handling

All samples were collected in EDTA tubes (BD vacutainer) and processed within 4 h. Human blood samples were centrifuged at 600 g for 5 min. Red blood cells were lysed in Ammonium-Chloride-Potassium (ACK) lysing buffer (150 mM NH_4_Cl, 12 mM NaHCO_3_, 1 mM EDTA, pH 7.4) in a ratio of 5 mL of blood for 20 mL of ACK buffer. After 5 min of incubation at room temperature, 25 mL of Phosphate Buffered Saline (PBS) were added and cells were centrifuged at 600 g for 5 min, and the supernatant was discarded. This procedure was repeated 3 times. Leukocytes were then resuspended in fluorescence-activated cell sorting (FACS) buffer (PBS 1 % BSA, 0.01 % NaN_3_, 1 mM EDTA) and prepared for flow cytometry (see below). Leukocyte count and viability (>95 %) were assessed by trypan-blue staining on a hemacytometer.

### 3.3 Flow cytometry staining

Non-specific antibody binding sites were saturated with 20 μL of a solution containing 100 μg/mL of human, mouse, rat, and goat IgG (Jackson ImmunoResearch Europe and Innovative Research Inc.) in FACS buffer. 100 μL of staining solution containing the mix of fluorophore-conjugated specific antibodies or their fluorophore-conjugated isotypes (Supplementary Table S2) were added to the cells for 30 min at 4 °C protected from light. After a wash in FACS buffer, cells were fixed in fixation buffer (Biolegend) for 20 min at 4 °C and then washed in FACS buffer before data acquisition. For phospho-protein flow cytometry procedures, 1.5 mL of 1X BD FACS Lysing Solution was added to 200 μL of whole blood in a 5 mL polypropylene tube (BD Falcon). After 10 min of incubation cells were washed in 2 mL of PBS, followed by a second wash in 2 mL FACS buffer. Cells were first stained extracellularly as described above, then washed in FACS buffer. Cell fixation and permeabilization was realized with the BD Cytofix/Cytoperm kit for 20 min at 4 °C. Cells were then washed in permeabilization/wash buffer, followed by intracellular staining performed in permeabilization/wash buffer for 30 min following the manufacturer's instructions. Cells were then washed and resuspended in FACS buffer before acquisition. Antibody panel 1: pSYK (Y525-526) Alexa Fluor (AF) 488, pSYK (Y348) PE, pPLCγ2 (Y759) PE/Vio770, CD19 BV605, CD27 PerCP/Cy5.5, CD45RA AF700, CD5 APC/Fire750, CD4 BV785, CD8α BV510, CD16 BV421 and CD14 PE/Dazzle 594. Panel 1 was used to quantify the four B cell subsets, the protein phosphorylations, CD4 and CD8 T cells, monocyte subsets, eosinophils and neutrophils. Antibody panel 2 for the quantification of basophils: CD14 APC/Fire750, CD16 BV421, CD19 BV605, CRTH2 AF647 and FcεRIα PE/Cy7. Antibody Panel 3 for the detailed B cell analysis: CD14 APC/Fire750, CD15 PE, CD16 AF700, CD19 BUV395, IgD PE/Cy7, CD27 PerCP/Cy5.5, CD24 AF647, CD95 BV785, CD11c PE/Dazzle594 and CXCR5 BV421. Flow cytometry acquisitions were carried out using a 4 lasers Becton Dickinson LSR Fortessa (panels 1 and 2) and a 5 lasers BD LSR Fortessa X20 (panel 3). Data analysis was realized with Flowjo v10.10.0 software (Treestar, BD Biosciences).

### 3.4 FACS-cell sorting

Cell sorting of monocyte subsets was performed using the Becton Dickinson FACSMelody cell sorter. Before cell sorting, cells were stained extracellularly as described above. Purity of isolated cells was checked after each sort and was comprised between 96 % and 99 %.

### 3.5 RNA extraction, reverse transcription, and real-time PCR

Lysis of FACS-sorted cells was realized using RLT buffer (Qiagen) supplemented by 1 % of β-mercaptoethanol. RNA extraction was performed with RNeasy micro kit (Qiagen) following the manufacturer's instructions. cDNA was obtained by reverse transcription using Superscript III first-strand synthesis system (Invitrogen) following the manufacturer's instructions. For real-time PCR (RT-PCR), genes of interest were targeted with the following pairs of primers: *TNF* Reverse (Rev) = 5′-GAG GAC CTG GGA GTA GAT GAG-3’/Forward (For) = 5′-CCT CTC TCT AAT CAG CCC TCT G-3’; *TNFSF13B* Rev = 5′-ATG TCC CAT GGC GTA GGT CT-3’/For = 5′-TGC AGA CAG TGA AAC ACC AAC T-3’; *IL1B*: Rev = 5′-CGT TAT CCC ATG TGT CGA AGA A-3’/For = 5′-AGC TAC GAA TCT CCG ACC AC-3’. RT-PCR reactions were performed using SsoAdvanced Universal SYBR Green Supermix (Bio-Rad) and amplification was measured by CFX 96 system (Bio-Rad).

### 3.6 Statistical analyses

Statistical analyses were performed using Prism 10.2.2 software (GraphPad). D'Agostino-Pearson omnibus normality tests were used to evaluate gaussian distributions. If values within compared groups assumed a normal distribution, one-way analyses of variance (ANOVA) followed by Tukey's multiple comparisons tests were performed. If at least one group of individuals did not comprise data assuming a gaussian distribution, Kruskal-Wallis tests followed by a Dunn's multiple comparisons test were applied. In Fig. 4, Fig. 2 different variables were compared between two groups. Analyses were by two-way ANOVA followed by uncorrected Fisher's tests. ∗*P* ≤ 0.05, ∗∗*P* ≤ 0.01, ∗∗∗*P* ≤ 0.001, ∗∗∗∗*P* ≤ 0.0001, ns = not significant. Factorial Discriminant Analysis (FDA) (Fig. 5) was performed using XLSTAT v2023.3.0 (Lumivero). FDA method was chosen to determine the most relevant quantitative variables (n = 12) for group discrimination (n = 6).

## Data sharing statement

All data reported in this paper will be shared by the corresponding author upon reasonable request.

## Declaration of competing interest

The authors declare the following financial interests/personal relationships which may be considered as potential competing interests:Eric Daugas reports financial support was provided by 10.13039/501100001665French National Research Agency. Nicolas CHARLES reports financial support was provided by 10.13039/501100001665French National Research Agency. Nicolas CHARLES reports financial support was provided by 10.13039/501100002915Foundation for Medical Research. If there are other authors, they declare that they have no known competing financial interests or personal relationships that could have appeared to influence the work reported in this paper.

## Data Availability

Data will be made available on request.
