# Supplementary material for: Immune characterization of lupus nephritis patients undergoing dialysis
Source: J Transl Autoimmun. 2025 May 2;10:100290. doi: 10.1016/j.jtauto.2025.100290 (PMC12124682; doi:10.1016/j.jtauto.2025.100290)
Supplement: Multimedia component 1 [file mmc1.docx]

**Immune characterization of lupus nephritis patients undergoing dialysis.**

**SUPPLEMENTARY MATERIAL**

**Authors**: Quentin Simon, PhD^a,b^; François Gaillard, MD,PhD^c^; John Tchen, PhD^a^; Delphine Bachelet, PhD^e^; Karim Sacré, MD, PhD^a,d^; Katell Peoc’h, PharmD, PhD^f,g^; Noémie Jourde-Chiche, MD, PhD^h,i^; Eric Daugas, MD, PhD^a,c*^; Nicolas Charles, PhD^a* ✉^.

**Affiliations**: **a**. Université Paris Cité, Centre de recherche sur l’inflammation, INSERM UMR1149, CNRS EMR8252, Laboratoire d’Excellence Inflamex, Paris, France. ; **b**. Inovarion, Paris, France. ; **c**. Department of Nephrology, Hôpital Bichat, Assistance Publique-Hôpitaux de Paris, Paris, France. ; d. Department of Internal Medicine, Hôpital Bichat, Assistance Publique-Hôpitaux de Paris, Paris, France. ; **e**. Department of biostatistical Epidemiology and clinical research, Hôpital Bichat, Assistance Publique-Hôpitaux de Paris, INSERM CIC-EC 1425, Paris, France. ; **f.** Université Paris Cité, Centre de Recherche sur l’Inflammation, INSERM UMR1149, Laboratoire d'Excellence GR-Ex, Paris, France. ; **g**. Service de Biochimie, Hôpital Bichat, DMU BIOGEM, Assistance Publique-Hôpitaux de Paris, Paris, France. ; **h**. Aix-Marseille Université, C2VN, INSERM, INRAE, Marseille, France. ; **i**. Assistance Publique-Hôpitaux de Marseille, Centre de Néphrologie et Transplantation Rénale, Hôpital de la Conception, Marseille, France.

*: ED and NC contributed equally to this work

Correspondence should be addressed to:

Nicolas Charles: nicolas.charles@inserm.fr. ORCID: [0000-0002-5416-5834](https://orcid.org/0000-0002-5416-5834). Centre de recherche sur l’inflammation, INSERM UMR1149, CNRS EMR8252, Université Paris Cité, Faculté de Médecine Site Bichat, 16 rue Henri Huchard, 75018 Paris, France. Tel. : +33157277306

**Keywords**: systemic lupus erythematosus, lupus nephritis, end stage kidney disease, dialysis, immunophenotyping, disease activity.

**Supplementary Figure S1**


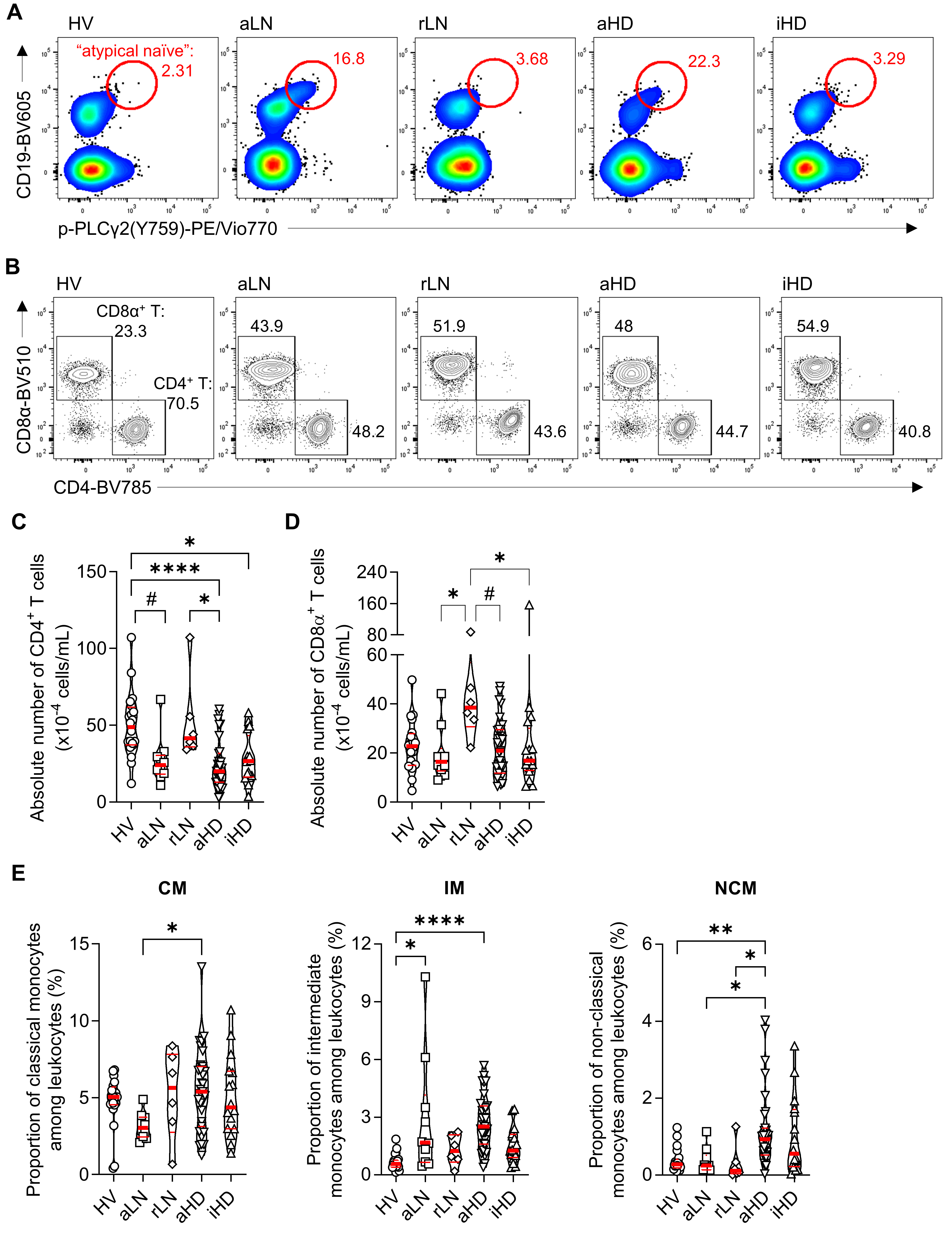


**Fig. S1: Atypical naive B cells, T cells and monocytes in the ELUDIAL cohort.**

(**A**) Selection of “atypical naive” B cells based on CD19 expression and p-PLCγ2(Y759) levels in SSC^lo^ CD16^–^ CD14^–^ leukocytes. Flow cytometry plots are representative of HV, aLN, rLN, aHD and iHD groups (same individuals as in (**Fig. 1A**)). Proportions (%) of “atypical naive” B cells among CD19^+^ B cells are indicated in red. (**B**) Representative flow cytometry plots of CD4 and CD8α expression in SSC^lo^, CD16^–^, CD14^–^, CD19^–^ and CD5^+^ leukocytes. Proportions (%) of CD4 and CD8α T cells are indicated next to the corresponding gates for HV, aLN, rLN, aHD and iHD individuals. Absolute numbers of CD4^+^ (**C**) and CD8α^+^ (**D**) T cells in HV (n=20), aLN (n=10), rLN (n=6), aHD (n=31) and iHD (n=16) groups. (**E**) Proportions (%) of classical monocytes (CM), intermediate monocytes (IM) and non-classical monocytes (NCM) among leukocytes in HV (n=20), aLN (n=10), rLN (n=6), aHD (n=31) and iHD (n=16) groups. (**C-E**) Data are presented as individual values in truncated violin plots showing median (thick red line) and quartiles (thin red lines). **P*≤0.05, ***P*≤0.01, *****P*≤0.0001, ns=not significant by Kruskal-Wallis tests followed by Dunn's post-tests. (**C**) # (HV *vs* aLN): *P*=0.0651, (**D**) # (rLN *vs* aHD): *P*=0.0588. Pairwise comparisons not displayed on graphs were ns.

**Supplementary Figure S2**


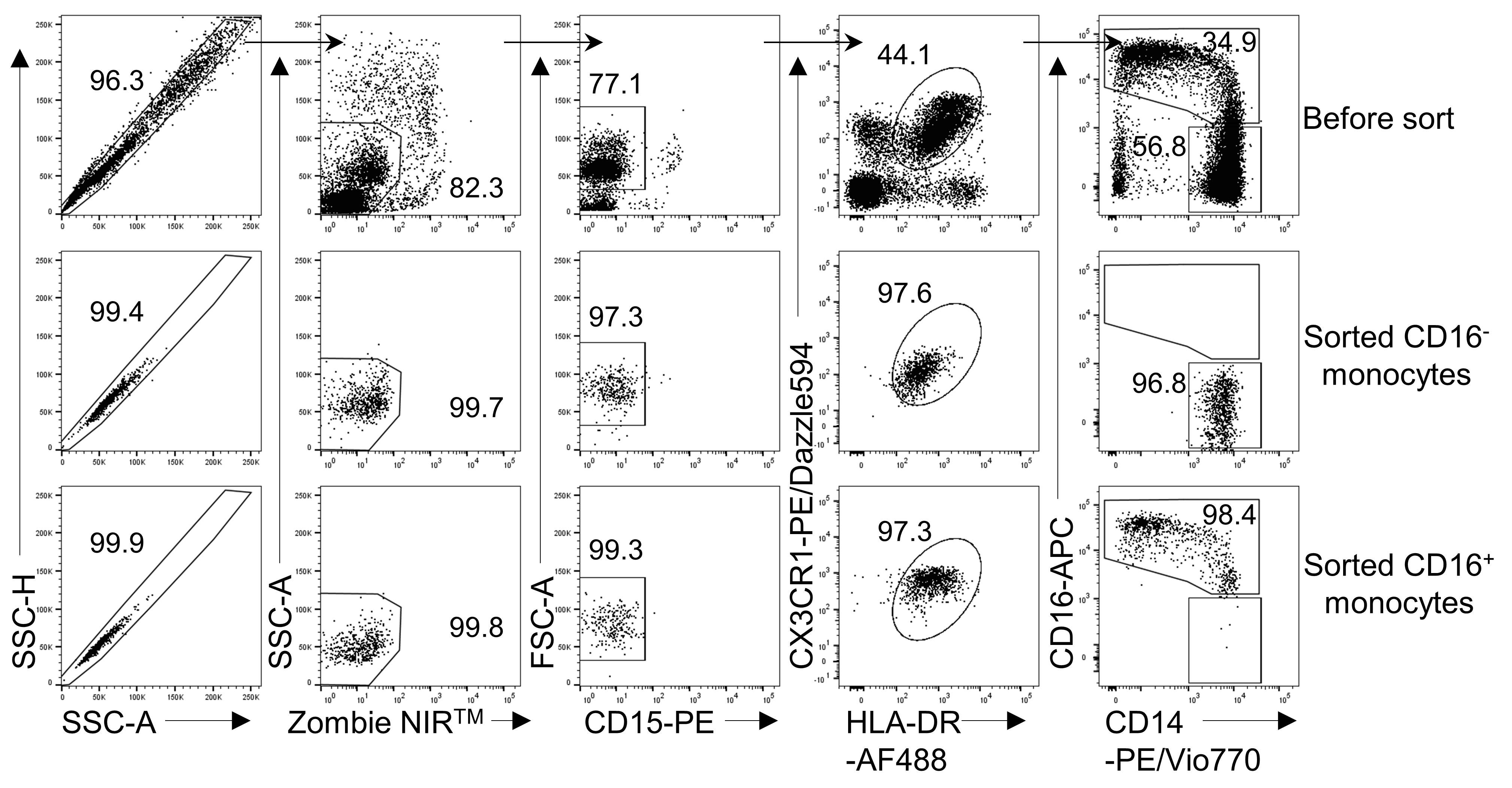


**Fig. S2. Purity of CD16^–^ and CD16^+^ monocytes isolated by fluorescence-activated cell sorting**.

Representative flow cytometry plots of gating strategy and purity of FACS-sorted CD16^–^ and CD16^+^ monocytes (related to **Fig. 4F**).

**Supplementary Figure S3**


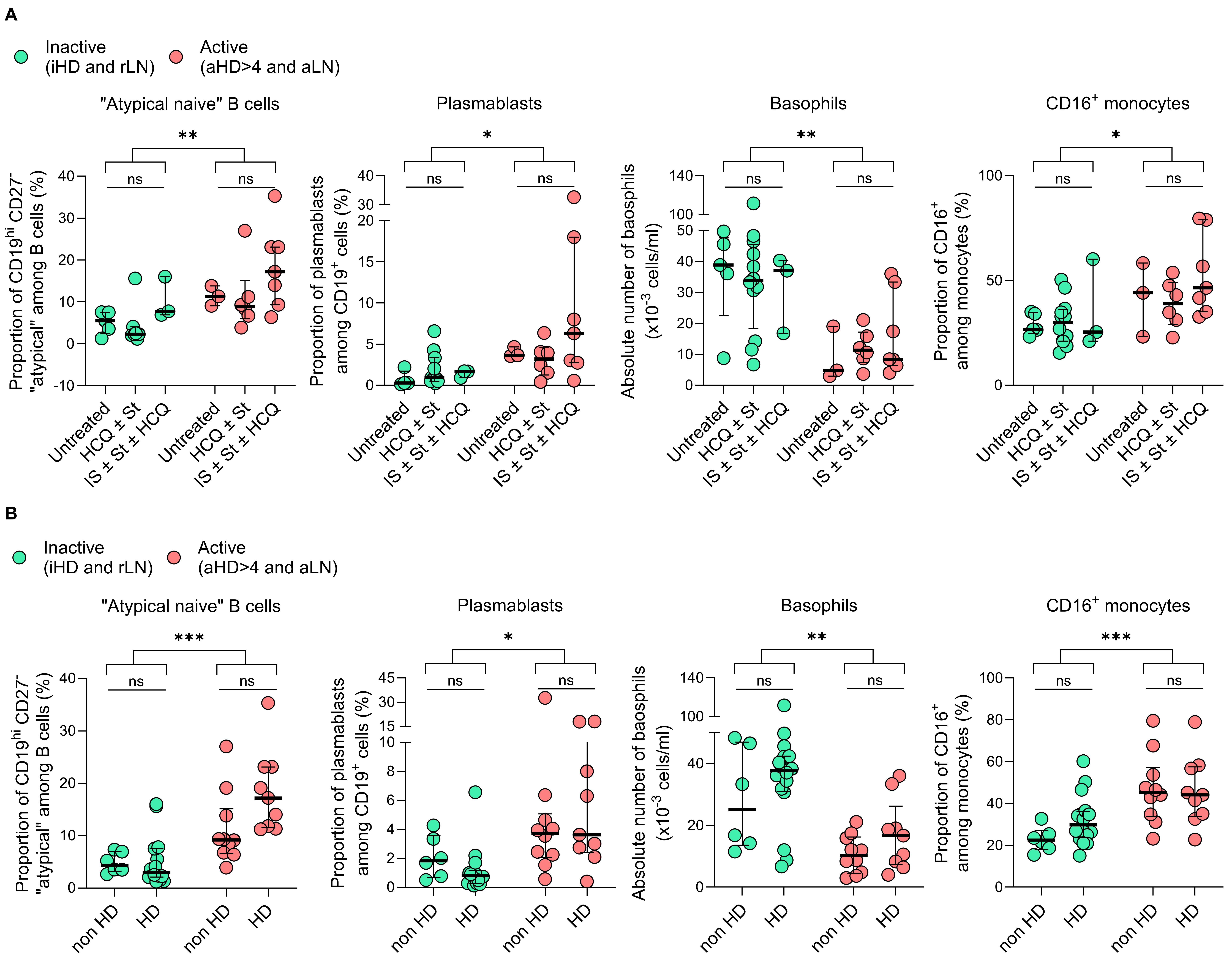


**Fig. S3. Lack of influence of treatments and dialysis therapy on the immune phenotype of SLE patients**.

(**A**) Effects of treatments and disease activity on “atypical naive” B cells (% of CD19^+^), plasmablasts (% of CD19^+^), basophils (counts) and CD16^+^ monocytes (% of monocytes) analyzed by two-way ANOVA showing an effect of the activity parameter but none of the treatment parameter on the populations. (HCQ: hydroxychloroquine, St: steroids, IS: immunosuppressive drugs). (**B**) Effects of dialysis and disease activity on the same parameters as in (**A**) analyzed by two-way ANOVA showing an effect of the activity parameter but none of the dialysis parameter on the populations. HD: hemodialysis. The inactive group was composed of iHD (n=16) and rLN (n=6) patients while the active group included aHD>4 (n=9) and aLN (n=10) SLE patients. Data are presented as individual values with their median and interquartile ranges. **P*≤0.05, ***P*≤0.01, ****P*≤0.001, ns=not significant. For plasmablasts analysis, 2 outliers were identified by ROUT test (1%) and removed from the inactive group.

**Supplementary Table S1**

 **Table S1. Individual demographic and clinical characteristics of SLE patients undergoing hemodialysis.**

#: patient number, iHD: inactive patients undergoing hemodialysis (HD), aHD≤4: active HD patients with nr (non-renal) SLEDAI ≤4, aHD>4: active HD patients with nrSLEDAI >4, SLEDAI: Systemic Lupus Erythematosus Activity Index, M: Male, F: Female, C3-4: Complement component 3-4, CH50: Complement Haemolytic activity. n: number, m: median, Y: Yes, N: No, POS: Positive, NEG: Negative, NA: Not Applicable.

**Supplementary Table S2**


**Table S2. List of antibodies, reagents, software, and equipment.**
